# Diagnosis and Management of Fetal Arrhythmias in the Current Era

**DOI:** 10.3390/jcdd11060163

**Published:** 2024-05-24

**Authors:** Stacy A. S. Killen, Janette F. Strasburger

**Affiliations:** 1 Thomas P. Graham Jr. Division of Pediatric Cardiology, Department of Pediatrics, Vanderbilt University Medical Center, Monroe Carell Jr. Children’s Hospital at Vanderbilt, 2200 Children’s Way, Suite 5230, Nashville, TN 37232, USA; 2 Division of Cardiology, Departments of Pediatrics and Biomedical Engineering, Children’s Wisconsin, Herma Heart Institute, Medical College of Wisconsin, Milwaukee, WI 53226, USA; jstrasburger@childrenswi.org

**Keywords:** fetal arrhythmias, fetal bradycardia, fetal tachycardia, fetal anti-Ro/SSA-mediated heart block, fetal long QT syndrome, fetal echocardiography, fetal magnetocardiography

## Abstract

Diagnosis and management of fetal arrhythmias have changed over the past 40–50 years since propranolol was first used to treat fetal tachycardia in 1975 and when first attempts were made at in utero pacing for complete heart block in 1986. Ongoing clinical trials, including the FAST therapy trial for fetal tachycardia and the STOP-BLOQ trial for anti-Ro-mediated fetal heart block, are working to improve diagnosis and management of fetal arrhythmias for both mother and fetus. We are also learning more about how “silent arrhythmias”, like long QT syndrome and other inherited channelopathies, may be identified by recognizing “subtle” abnormalities in fetal heart rate, and while echocardiography yet remains the primary tool for diagnosing fetal arrhythmias, research efforts continue to advance the clinical envelope for fetal electrocardiography and fetal magnetocardiography. Pharmacologic management of fetal arrhythmias remains one of the most successful achievements of fetal intervention. Patience, vigilance, and multidisciplinary collaboration are key to successful diagnosis and treatment.

## 1. Introduction

Fetal arrhythmias complicate at least 1–3% of pregnancies and account for about 20% of referrals to fetal cardiologists [1]. However, this likely significantly underestimates the true burden of the disease, as “silent arrhythmias” may explain even more fetal demises (up to 10%), in utero hydrops, and premature delivery [2]. Ten percent of fetal arrhythmias are known to be life-threatening [1]. But markers of life-threatening arrhythmias may go unrecognized [3]. The most lethal abnormalities in cardiac conduction may not manifest as changes in heart rate or rhythm and, thus, may not be apparent by ultrasound or fetal echocardiography [3]. Pharmacologic management of fetal arrhythmias remains one of the most successful achievements of fetal intervention [4], but not all therapies are effective. While the identification and management of fetal arrhythmias have improved over the past 40–50 years, there is yet more to be achieved in diagnostic tools and therapeutic options. Importantly, care of mothers and babies affected by fetal arrhythmias involves a multidisciplinary team that must understand the effect of treatment on both mother and baby [5]. Obstetrical providers are on the frontlines of identifying fetal arrhythmias and must be supported by perinatologists, cardiologists, and neonatologists. This article will review the types of fetal arrhythmias and discuss diagnosis and management in the current era.

## 2. Types of Fetal Arrhythmias

There are three broad categories of fetal arrhythmias: extrasystoles (early extra beats from the atria or ventricles), tachycardias (abnormally fast heart rates), and bradycardias (abnormally slow heart rates). Pregnancies may be complicated by just one type of arrhythmia or by combinations of irregularities in the heart rate and rhythm. The normal fetal heart rhythm is regular and characterized by one-to-one conduction from the atria to the ventricles. The normal fetal heart rate (FHR) is gestational-age-dependent but has generally been defined by obstetricians as a rate between 120–160 beats per minute (bpm) and by fetal cardiologists as a rate between 110–180 bpm [6,7]. A 5–15 bpm FHR variation is expected in healthy fetuses; fetal movements, as characterized by actigraphy, are associated with these heart rate changes [1]. Over the past 15 to 20 years, fetal cardiologists and obstetricians have become increasingly aware of the shortcomings of these definitions, with much research demonstrating that “subtle” arrhythmias may be missed with these broad heart rate limits [3,8,9]. Persistent FHR elevations just above the upper limit of “normal” may go unrecognized as markers of fetal thyrotoxicosis, for example [10,11]. Even more problematic are FHRs within the “normal” range that mask potentially lethal channelopathies, like long QT syndrome (LQTS) [8,9]. Mitchell et al. have further characterized FHRs at the 3rd, 50th, and 97th percentile for gestational age and emphasized the importance, for both mother and fetus, of recognizing FHRs persistently <3rd percentile for gestational age [8].

## 3. Extrasystoles

Early extra beats from the atria or ventricles—premature atrial (PACs) or ventricular (PVCs) contractions—are thought to be the most common type of fetal arrhythmia [3,12]. Premature junctional contractions (PJCs) can also occur but are more difficult to identify by ultrasound. While PACs are generally considered to be a benign arrhythmia of the immature fetal electrical system, PVCs are more commonly associated with cardiac structural, functional, or electrical disease [13,14]. Distinguishing the two by ultrasound is possible but sometimes challenging.

**Figure 1:**
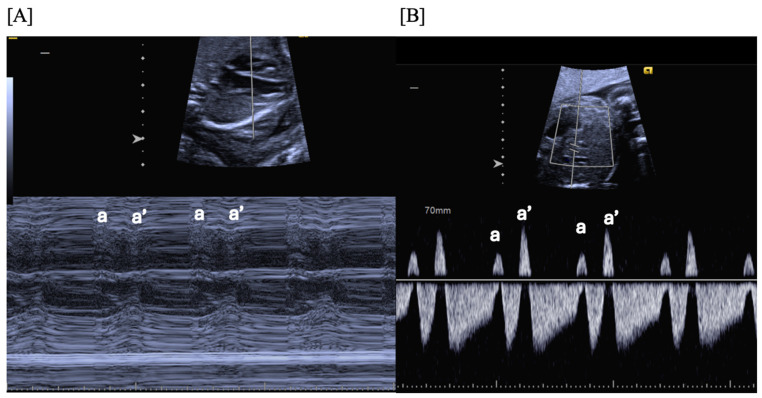
Fetal blocked atrial bigeminy, in which the sinus node atrial contraction (a) is conducted to the ventricle, while the subsequent premature atrial contraction (a’) is not conducted to the ventricle in a patterned fashion. M-mode (**A**) and hepatic vein Dopplers (**B**) demonstrate a short–long–short–long a–a’ interval.

Atrial ectopy (PACs) can be intermittent or regular and may be conducted to the ventricles or blocked. PACs are characterized by an early atrial contraction and less than a full compensatory pause [12,15]. The most common abnormality of fetal heart rhythm (1:400 pregnancies and 2% of 2nd- and 3rd- trimester pregnancies), PACs are usually benign, with no associated hemodynamic compromise, even when they are very frequent [2]. They usually resolve spontaneously in utero or in the early postnatal period. In utero or neonatal supraventricular tachycardia (SVT) can occur in 0.5–2% of fetuses presenting with PACs [3]. The risk of SVT is increased to about 10% when complex ectopy is noted or when the ectopy is re-entrant, with a fixed interval between the normal beat and the early beat and with a bigeminal (blocked atrial bigeminy, Figure 1) or trigeminal pattern [3]. In about 1% of cases, PACs are associated with congenital heart disease, myocarditis, LQTS, cardiomyopathy, or cardiac tumors, like rhabdomyomas [15]. 

Ventricular ectopy (PVCs) can be distinguished from PACs by earlier contraction of the ventricle rather than the atrium, a regular atrial rate, dissociation of the ventricular contraction from the atrial contraction, and a full post-ectopic compensatory pause in the ventricular-ventricular (VV) interval; there may be associated atrioventricular valve insufficiency on ultrasound [12,15]. PVCs are less common than PACs and are more typically associated with myocarditis, LQTS or other channelopathies, cardiomyopathy, cardiac tumors, or third-degree atrioventricular block, especially when escape rates are <55 bpm [13].

Fetuses with isolated extrasystoles should not demonstrate signs of heart failure. Because of the potential risk of developing SVT in the setting of PACs, especially patterned PACs, some centers recommend frequent obstetrical heart rate monitoring for about 4–5 weeks after diagnosis—until the PACs resolve or until delivery, whichever comes first [12]. But variation in care exists among obstetrical providers and fetal cardiologists. Some, but not all, recommend postnatal EKG and echocardiogram. Delivery at a tertiary care center is rarely indicated. However, mothers with fetal PVCs are often recommended to deliver at a center where postnatal evaluation for baby can include an EKG, echocardiogram, and at least 24 h of continuous telemetry monitoring [3,13]. Further evaluation and follow-up, often in concert with an electrophysiologist, is based on the results of these initial studies.

## 4. Tachyarrhythmias

Fetal tachyarrhythmias complicate 0.5% of pregnancies and are associated with high morbidity and mortality [13,16,17]. They are one of the few fetal cardiac emergencies. Untreated tachyarrhythmias can cause in utero cardiomyopathy, cardiac failure, hydrops, premature delivery, and fetal demise [16]. Sustained fetal tachycardia with hydrops has a mortality rate as high as 30–50% [18,19], but in the absence of hydrops, tachycardia-related perinatal mortality is much lower (4%) [20]. In utero antiarrhythmic therapy can restore sinus rhythm but poses significant risks to mother and fetus, including fatal pro-arrhythmia [18]. Delivery of a premature, hydropic fetus may be required, with associated high mortality and morbidity, including neurological sequelae [16,20,21,22].

Fetal tachycardia is generally defined by fetal cardiologists as FHR ≥ 180 bpm [12]. However, sustained and/or unvarying FHRs ≥ 160–170 bpm may also be abnormal and warrant treatment [23]. Fetal tachycardias may originate from the sinus node (sinus tachycardia), from the atria (atrial tachycardia or atrial flutter), from an accessory pathway (reentrant tachycardia), or, rarely, from the ventricles (ventricular tachycardia) [12]. Associated congenital heart disease occurs in ~5–10% of fetuses with SVT [1,20,24]. However, in most fetuses with SVT, the reason tachycardia develops is unknown; one study found that maternal thyroid disease was more common in fetuses with SVT compared with controls (odds ratio = 9.8, 95% confidence interval 2.3–42.3) [25].

**Figure 2:**
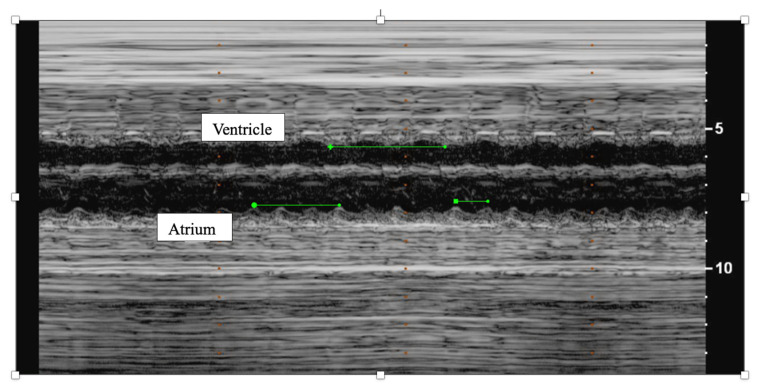
M-mode of fetal atrial flutter demonstrating twice as many atrial contractions (atrium) as ventricular contractions (ventricle).

There are four primary mechanisms that produce SVT (Table 1):

- *Atrioventricular re-entrant tachycardia (AVRT)* involving the atrioventricular (AV) node for antegrade conduction and a fast retrograde-conducting accessory AV pathway;
- *Permanent junctional reciprocating tachycardia (PJRT)* with re-entry across a concealed slow retrograde-conducting accessory pathway;
- *Ectopic atrial tachycardia (EAT)* due to enhanced automaticity of atrial tissue;
- *Atrial flutter (AF)* due to a macro-re-entrant pathway within the atrial myocardium and associated with varying degrees of AV block (Figure 2).

**Table 1:** Characteristics of the various types of fetal supraventricular tachycardia (SVT), including typical gestational age (GA) at diagnosis, usual rates, onset and termination of tachycardia, and atrioventricular (AV) relationships. VA, ventricular–atrial; VV, ventricular–ventricular; AVRT/ORT, atrioventricular reentrant tachycardia/orthodromic reciprocating tachycardia; AVNRT, atrioventricular nodal reentrant tachycardia; EAT, ectopic atrial tachycardia; PJRT, permanent junctional reciprocating tachycardia.

	Short VA SVT (60–70%) *AVRT/ORT* *AVNRT*	Long VA SVT *EAT* *PJRT*	Atrial Flutter (25–30%)
	“Faster and Later”	“Slower and Earlier”	“Variable AV Conduction”
**Typical GA at ** **Diagnosis**	>18 weeks GA	>12 weeks GA	>28 weeks GA
**Usual Rates**	210–320 bpm	170–220 bpm	Atrial rate 300–550 bpm Ventricular rate 180–240 bpm
**Onset and ** **Termination**	- Sudden onset/offset - Blocks in the AV node and terminates with a non-conducted atrial contraction	- Gradual onset and offset - Terminates with a non-conducted ventricular contraction (EAT)	70% of fetuses with atrial flutter have accessory pathways and may have ORT postnatally
**Atrioventricular (AV) Relationship**	- 1:1 - VA interval < ½ of the VV interval - VA/AV ratio < 1	- 1:1; may be variable (EAT) - VA interval > ½ of the VV interval - VA/AV ratio > 1	Variable degrees of AV block (primarily 2:1 or 3:1); fixed ventricular rate

AVRT (60–70%) and atrial flutter (30%) are the most commonly observed SVT types [20,24,26].

SVT can also be categorized into “short” and “long” VA tachycardia based on the relationship of atrial and ventricular contractions [12,27,28]; this distinction has implications for therapy.

**Figure 3:**
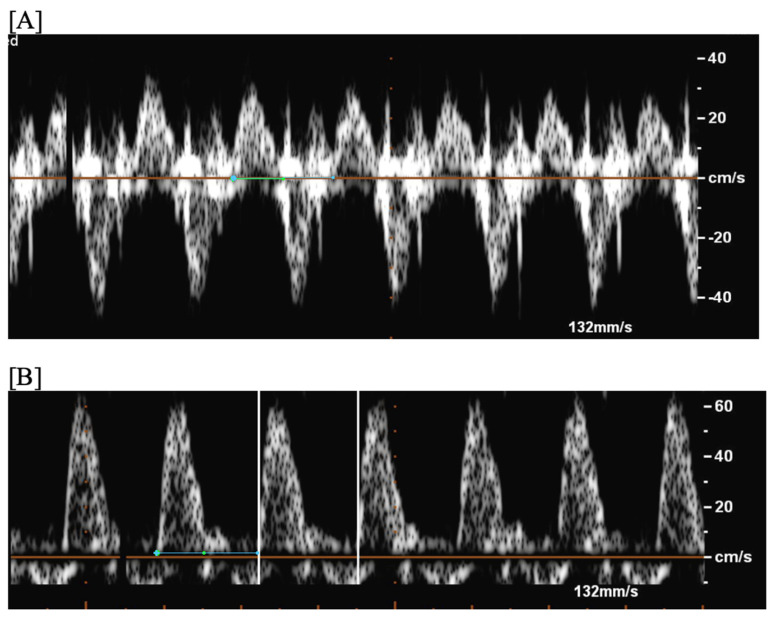
Fetal short VA tachycardia: simultaneous mitral inflow–aortic outflow Doppler (**A**) and simultaneous superior vena cava–aortic (SVC-Ao) outflow Doppler (**B**).

**Figure 4:**
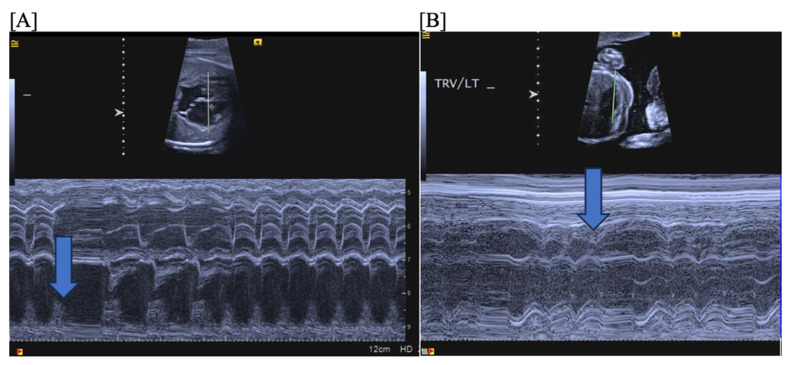
Fetal short VA tachycardia with termination (as noted by the blue arrow in the figures) in the atrium (**A**) and long VA tachycardia with termination in the ventricle (**B**).

- Short VA SVT (Figure 3 and Figure 4) demonstrates a ventricular–atrial (VA) interval that is less than half of the ventricular–ventricular (VV) interval (VA:AV ratio < 1) and a sudden onset and termination of tachycardia; tachycardia usually terminates with a non-conducted atrial contraction. Short VA SVT includes AVRT (also known as orthodromic reciprocating tachycardia, ORT) and atrioventricular nodal reentrant tachycardia (AVNRT). Short VA SVT typically presents after 18 weeks of gestation.
- Long VA SVT (Figure 4) demonstrates a VA interval that is more than half of the VV interval (VA:AV ratio > 1). Long VA SVT includes EAT and PJRT. A distinguishing feature of EAT is tachycardia termination with ventricular contraction. Long VA SVT may occur as early as 12 weeks of gestation. Because long VA tachycardias have slower rates, they are less likely to cause hydrops.

Fetal SVT may be sustained or intermittent; tachycardia duration has implications for therapy. Sustained/incessant SVT represents >12 h of uninterrupted tachycardia or tachycardia present ≥50% of the echocardiographic monitoring time (typically ~30 min) [3,18,29]. Intermittent SVT is present ≤50% of the echocardiographic monitoring time or in <12 h of a 24 h fetal monitoring period [3,18,29].

In total, 40% of fetuses with SVT have heart failure/hydrops at presentation [19,20]. Risk factors for developing hydrops include sustained/incessant SVT, AVRT as the tachycardia mechanism, faster ventricular rates, onset of tachycardia before 32 weeks of gestation, and co-occurrence of structural heart disease [19,26].

**Figure 5:**
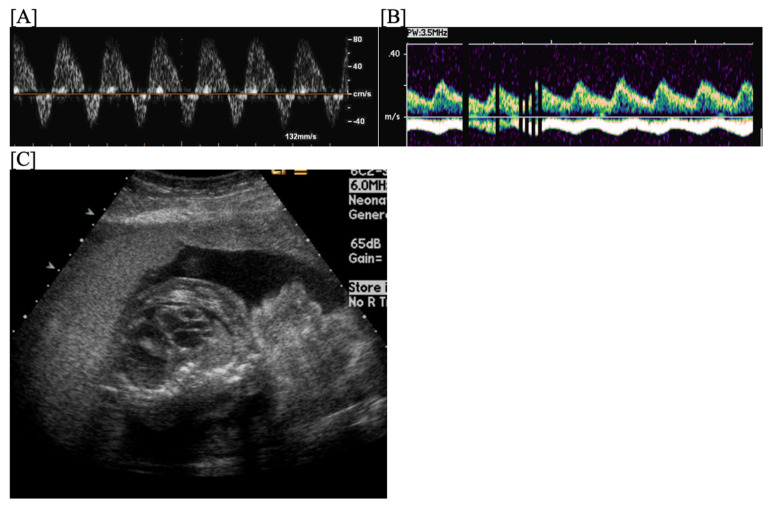
Fetal short VA tachycardia: to-fro ductus venosus pattern, which is characteristic of short VA tachycardia (**A**); there are pulsations in the umbilical vein (**B**) in the setting of fetal hemodynamic compromise and hydrops (**C**).

Fetal tachycardia type and associated signs of hemodynamic compromise (hydrops, cardiomegaly, atrioventricular valve regurgitation, altered ductus venosus flow patterns, and ventricular dysfunction) are important determinants of outcome. The ductus venosus, which is often a sensitive marker of fetal well-being, is difficult to interpret during tachycardia, as it is typically abnormal when the fetal heart rate exceeds 210 bpm [30]. A to–fro flow pattern is usually observed in (and may be a diagnostic marker of) short VA tachycardia, which is also more commonly associated with hydrops (Figure 5). 

Fetuses with SVT are at risk for preterm delivery by cesarean section, with its associated morbidities and mortalities. Treatment of fetal SVT is conducted with the goal of conversion to normal sinus rhythm or with the goal of achieving a normal ventricular rate to increase cardiac output and decrease the risk of and/or resolve hydrops [29,31]. Successful cardioversion can facilitate term, vaginal delivery and should be the goal in the current era [32]. Intermittent SVT without hydrops or other signs of hemodynamic compromise may not require treatment but close fetal monitoring is recommended [29].

Gestational age at tachycardia presentation, tachycardia mechanism and duration, associated fetal hydrops/hemodynamic compromise, and maternal risk factors for tolerating therapy are important to consider when choosing whether to initiate fetal anti-arrhythmic therapy or to proceed with premature, Cesarean section delivery and when choosing the type of anti-arrhythmic therapy [29]. In a recent multicenter study, Holmes et al. evaluated 37 pregnancies presenting in sustained tachycardia ≥35 weeks of gestation and treated with transplacental anti-arrhythmic therapy [32]. In-utero treatment restored sinus rhythm in 35 (95%) fetuses at a median of 2 (range, 1–17) days, including three of four hydropic fetuses [32]. The authors concluded that in-utero treatment of the near term and term fetus with SVT is highly successful even in the presence of hydrops, allowing vaginal delivery closer to term and avoiding unnecessary Cesarean section [32].

Most types of fetal SVT are treatable; even in hydropic fetuses, survival rates of 80–90% are achievable with current approaches to therapy [19,23], but it is important to remember that both mother and fetus can be affected by the therapy. Current pharmaceutical agents are administered “off-label” via the maternal circulation (“transplacental therapy”) or directly into the fetus to treat fetal SVT. Propranolol was first used to treat fetal tachycardia in 1975 but is no longer thought to be efficacious at currently recommended maternal doses [29]. In the current era, the primary antiarrhythmic agents used for treating fetal SVT include digoxin, sotalol, and flecainide (Table 2) [19]. Amiodarone is typically reserved for refractory tachycardia with hydrops given its significant side effect profile [19,33]. Procainamide, verapamil, and quinidine are no longer recommended to treat fetal SVT [29,34].

In a retrospective multicenter study comparing non-randomized treatment to digoxin, sotalol, and flecainide, tachycardia mechanism and duration, presence of associated hydrops/fetal hemodynamic compromise, and choice of first-line therapy significantly influenced fetal response to therapy [19]. Digoxin or flecainide is currently considered a first-line therapy for reentrant SVT/AVRT [19,35], while sotalol is currently considered to be the best first-line therapy for fetal atrial flutter [19,36,37,38]. Because flecainide can increase the ventricular response and cause ventricular pro-arrhythmias, it is not usually used to treat atrial flutter [18]. Ectopic atrial tachycardia often responds best to flecainide or digoxin [29,35], while PJRT is often best treated with flecainide or sotalol [29,39]. EAT and PJRT can be more difficult to treat than AVRT. Treatment of a hydropic fetus usually requires combination therapy. The combination of digoxin and sotalol is thought to be more effective for treatment of atrial flutter in the setting of fetal hydrops, while digoxin and flecainide are thought to be the best combination for reentrant SVT/AVRT [19]. Two recent meta-analyses evaluating transplacental SVT therapies found that flecainide was more effective than digoxin at converting fetal reentrant SVT to sinus rhythm, especially in the setting of fetal hydrops [19,40,41]. A prospective, multi-center study (Fetal Atrial Flutter and Supraventricular Tachycardia (FAST) therapy trial; www.fasththerapytrial.com; https://clinicaltrials.gov/study/NCT02624765, all accessed on 19 May 2024) to assess safety and efficacy of these agents is currently underway and promises to add clarity to first-line therapy choice for fetal SVT and atrial flutter.

Amiodarone been shown to be effective for conversion of reentrant SVT, ventricular tachycardia, and junctional tachycardia but less effective for atrial flutter [42]. When this therapy is used, discontinuation of therapy after the fetus has had resolution of hydrops, sustained sinus rhythm for 3 consecutive weeks, and/or by 34–35 weeks of gestation is recommended to minimize postnatal complications [33]. Thyroid function tests are recommended for mother and baby at birth, 2 weeks of age, and then monthly for the first 3 months of life because of the risk of neonatal hypothyroidism [33,43,44].

Direct fetal treatment with digoxin or amiodarone can be performed with cordocentesis (risk for cardiac arrest and fetal demise from cord injury), intramuscular (preferred; to fetal thigh or buttock), or intraperitoneal injection [45,46]. This form of SVT therapy is typically reserved for refractory tachycardia in severely hydropic fetuses [46]. Rarely, direct administration of adenosine is used to terminate fetal tachycardia [47].

Prior to initiating fetal therapy, maternal assessment with EKG, +/−echocardiography, serum electrolytes, complete blood count, TSH/fT4, 25(OH)-vitamin D level, renal function, and liver function is recommended [23]; evaluation for anti-Ro/SSA antibodies is recommended for fetal atrial flutter due to its association with immune-mediated carditis [48,49]. Correction of underlying maternal electrolyte abnormalities (calcium, potassium, and magnesium) and treatment of vitamin D deficiency can facilitate pharmaco-conversion of fetal SVT and help maintain sinus rhythm once conversion occurs [2].

Treatment of fetal SVT is primarily transplacental (given orally or intravenously to the mother) and vigilant monitoring for fetal and maternal adverse events, including pro-arrhythmia, is important [34]. Pharmacologic therapy is usually initiated in the hospital because of the risk for life-threatening pro-arrhythmias. Inpatient monitoring often includes continuous cardiac monitoring for both mother and fetus for at least 48–72 h (5–6 doses of therapy), daily EKGs, frequent electrolyte evaluation, and periodic measurement of drug levels (especially for digoxin and flecainide) [34]. Typically, in a non-hydropic fetus, 48–72 h of observation to assess fetal response to a maternal therapy are recommended before trialing a different agent [19]. Once cardioversion or rate control is achieved, transplacental therapy is usually continued until delivery; sometimes, a lower maintenance dose can control tachycardia while minimizing side effects and pro-arrhythmia for both mother and fetus [34].

Monitoring fetuses after SVT conversion is important as fetal diuresis after SVT conversion can cause rapid, progressive polyhydramnios and preterm labor in up to 40% of fetuses [2]. Additionally, SVT and atrial flutter can recur in utero (rates as high as 44% for SVT and 26% for atrial flutter in the Miyoshi study) despite conversion to a normal ventricular rate and in the setting of maintenance therapy [31,50]. Home fetal heart rate monitoring using a handheld Doppler can detect recurrence of fetal tachycardia after successful conversion and is a maternally-empowering adjunct to in-office monitoring and fetal echocardiography [51].

Despite vigilant fetal and maternal monitoring, fetal deaths, even in non-hydropic fetuses, have been reported in virtually every published series of fetal SVT treatment [34]. These deaths have been reported with escalation of a drug dose but have also been observed after weeks of SVT control with a stable dose. This raises concern about the potential of various anti-arrhythmic agents to cause a new arrhythmia or worsen the existing arrhythmia [52]. Because sotalol and flecainide reach concentrations many times higher in amniotic fluid compared to in maternal serum, fetal toxicity can occur (through fetal swallowing of amniotic fluid) without maternal evidence of toxicity [53,54]. This emphasizes the importance of more universal access to fMCG, a tool that can measure fetal repolarization abnormalities (QRS widening, QTC prolongation, T wave abnormalities), especially during prolonged treatment exposures to flecainide, sotalol, or amiodarone [52]. Additionally, it is important when treating fetal tachycardia to use the lowest effective dose and monotherapy when possible [34].

Successful cardioversion of fetal SVT can facilitate term, vaginal delivery and should be the goal in the current era, but it is important to remember that SVT can recur postnatally even when in utero control is achieved [55]. There appear to be few prenatal predictive factors for postnatal recurrence of SVT. In a study by Moodley, et al., two thirds of fetuses with SVT demonstrated postnatal tachycardia, mostly within the first 48 h of life [56], and 44% of those who converted to sinus rhythm prenatally demonstrated SVT postnatally [56]. Hinkle et al. found a 61% postnatal recurrence rate of SVT in their cohort, with a strong correlation between postnatal SVT and a later gestational age at fetal SVT diagnosis (median EGA 30 vs. 27.5 weeks, *p* = 0.006) [57]. Strasburger, et al. reported that 50% of infants with fetal SVT require no antiarrhythmic treatment postnatally; encouragingly, even more outgrow the need for medications by 1 year of age [3,13,26,58]. Atrial flutter is less likely to occur postnatally than reentrant SVT; once sinus rhythm has been established, recurrence of atrial flutter is rare in the absence of congenital heart disease [36]. However, some children who have reentrant SVT postnatally had atrial flutter in utero [58,59]. Only a small percentage of children (~10%) with fetal tachycardia are found to have Wolff–Parkinson–White syndrome/pre-excitation postnatally [20,60]. Careful screening for postnatal SVT is recommended in all newborns who had SVT in utero.

*Sinus tachycardia* is also a type of long-VA tachycardia. It is usually characterized by FHRs of 160–200 bpm with preserved heart rate variability and 1:1 AV conduction. Etiologies include hyperthyroidism/fetal thyrotoxicosis, myocarditis, anemia, hypoxia, acidosis, and exposure to maternal stimulants [12]. Treatment involves addressing the underlying cause.

*Junctional (JET) and ventricular (VT) tachycardias* are less common and are sometimes misdiagnosed as supraventricular tachycardias, but differentiating them has important implications for therapy and survival [61,62]. Ventricular tachycardias are characterized by VA dissociation with a slower atrial rate and variable AV and VA intervals [14]. Treatment of VT depends on whether LQTS is suspected. While sotalol and amiodarone can successfully restore sinus rhythm for VT without LQTS, they can prolong the QTc interval and worsen torsades de pointes in fetuses with LQTS [34].

## 5. Bradyarrhythmias

While a sustained FHR < 110 bpm has traditionally been used to diagnosis fetal bradycardia, research from the last decade has revised the definition based on gestational age and has improved our recognition of fetal bradyarrhythmias [2]. Because FHRs are gestational-age dependent and decrease as gestation progresses, FHRs persistently <3rd percentile for gestational age more accurately identify conduction disease [8]. According to Winbo et al., as a marker for LQTS, a mean FHR ≤ 133 bpm in the third trimester has a high specificity (>97%) but low sensitivity (<50%) [63]. Sinus bradycardia is the arrhythmia most associated with long QT syndrome and has been the reason for diagnosis in ~40% of cases [64,65].

**Figure 6:**
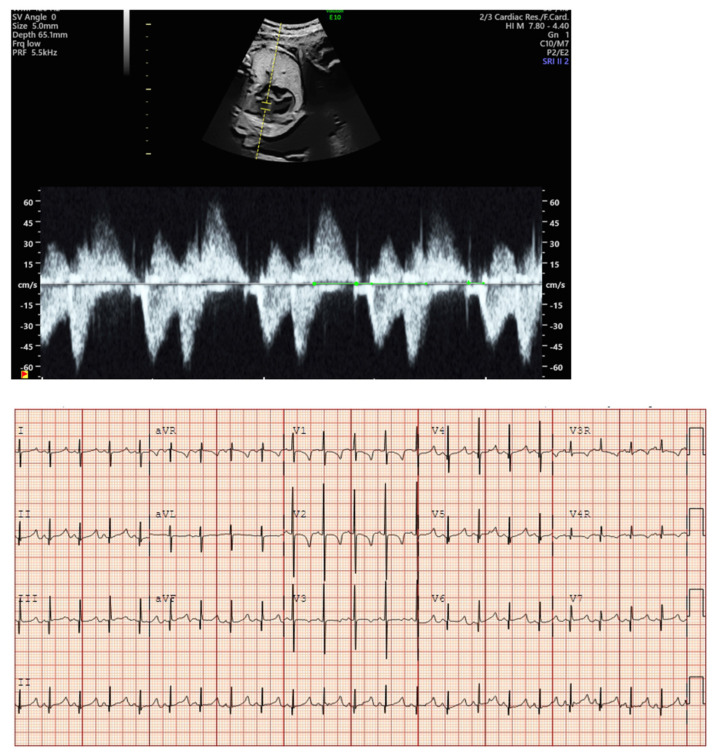
Fetal bradycardia (heart rate < 3rd percentile for GA) with first-degree AVB (prolonged mechanical PR interval) and postnatal EKG in a fetus with prenatal genetic testing by amniocentesis demonstrating a mutation in NKX2.5.

Sinus bradycardia is usually identified by persistent FHRs 90–130 bpm, regular atrial rates, and 1:1 AV conduction [2]. Etiologies for sinus bradycardia include, maternal hypothyroidism, effects from maternal medications (beta-blockers, anti-thyroid medications, methadone), anti-Ro/SSA mediated sinus node dysfunction, myocarditis, metabolic disorders (Pompe disease), Holt–Oram syndrome (*TBX5* mutations; associated with right atrial enlargement, limb abnormalities, sinus bradycardia, and first-degree atrioventricular block), NKX2.5 mutations (Figure 6), LQTS and other heritable channelopathies or inherited bradycardia syndromes, fetal CNS abnormalities, fetal severe growth restriction or distress, and umbilical cord compression (usually transient and iatrogenic from the ultrasound probe) [2,9,66]. No treatment is recommended for fetal sinus or low atrial bradycardia, but diagnostic evaluation and monitoring are recommended [2].

**Figure 7:**
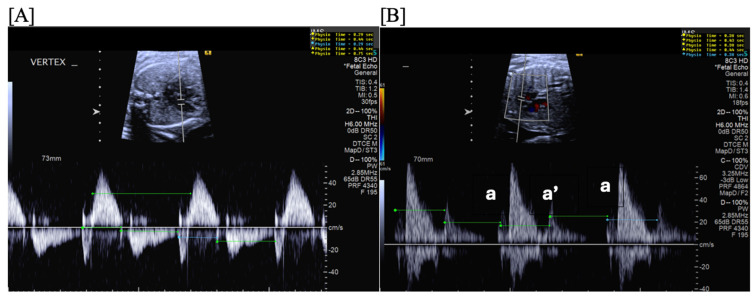
Fetal blocked atrial bigeminy with inflow-outflow (**A**) and SVC-Ao (**B**) Dopplers demonstrating the variable, patterned atrial rate characteristic of blocked atrial bigeminy.

Blocked atrial bigeminy (BAB) is a type of patterned premature atrial contractions in which every other atrial beat is non-conducted to the ventricles (Figure 7). This bradyarrhythmia is characterized by irregular atrial rates, with alternating short and long atrio-atrial (a-a’) intervals, and slower ventricular rates (60–90 bpm) than in sinus bradycardia [67]. While blocked atrial bigeminy is a benign arrhythmia, it increases the risk for developing supraventricular tachycardia to ~10% [12,15]. A postnatal EKG is recommended; for blocked atrial bigeminy that persists to delivery, 24 h continuous telemetry monitoring or 24 h Holter monitor after birth evaluates the risk for ectopic atrial tachycardia [15].

**Figure 8:**
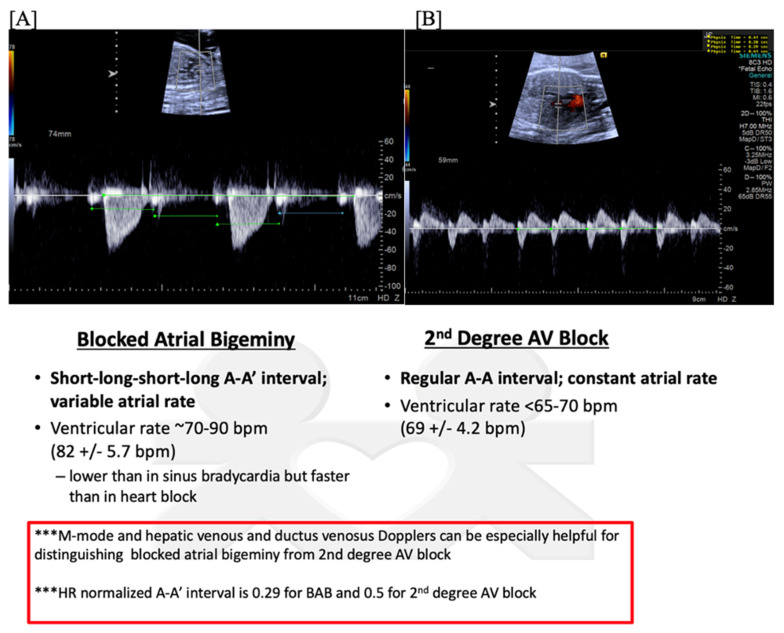
Fetal second-degree AVB demonstrated by SVC-Ao Doppler (**A**), with hepatic vein Doppler (**B**) confirming a regular atrial rate. Distinguishing features of blocked atrial bigeminy and second-degree AVB.

Second (2°)- or third (3°)-degree atrioventricular (AV) block is differentiated from blocked atrial bigeminy by a regular atrial rate with AV dissociation and slower ventricular rates (50–70 bpm); this is often best appreciated by hepatic venous Dopplers (Figure 8). The heart rate normalized a-a’ interval is 0.29 for BAB and 0.5 for 2°-AV block [68]. The isovolumic contraction time may be prolonged in 2°-AV block but not in BAB [67]. Etiologies of AV block include autoimmune-mediated (maternal anti-Ro/SSA antibodies), non-immune-mediated, congenitally corrected transposition of the great arteries (*l*-looped ventricles), heterotaxy syndromes (especially left atrial isomerism), and transient, benign AV block [69,70]. Third-degree AV block with associated complex congenital heart disease (ccTGA, left atrial isomerism) is associated with a mortality of ~100% in the presence of hydrops [69].

### 5.1. Long QT Syndrome and Other Channelopathies

The most lethal cardiac rhythm disturbances occur during normal rate and regular rhythm and are due to depolarization and repolarization abnormalities [71]. Channelopathy gene mutations have reportedly been found in 8.8% of intrauterine fetal demise and ~10% of sudden infant death [65,72,73]; however, recent data suggest that LQTS is a rare cause of sudden infant death syndrome [74]. Fetal heart rates < 3rd percentile for gestational age and a family history of fetal/neonatal demise or sudden unexplained death are keys to identifying these “silent” arrhythmias [8,64]. 

**Figure 9:**
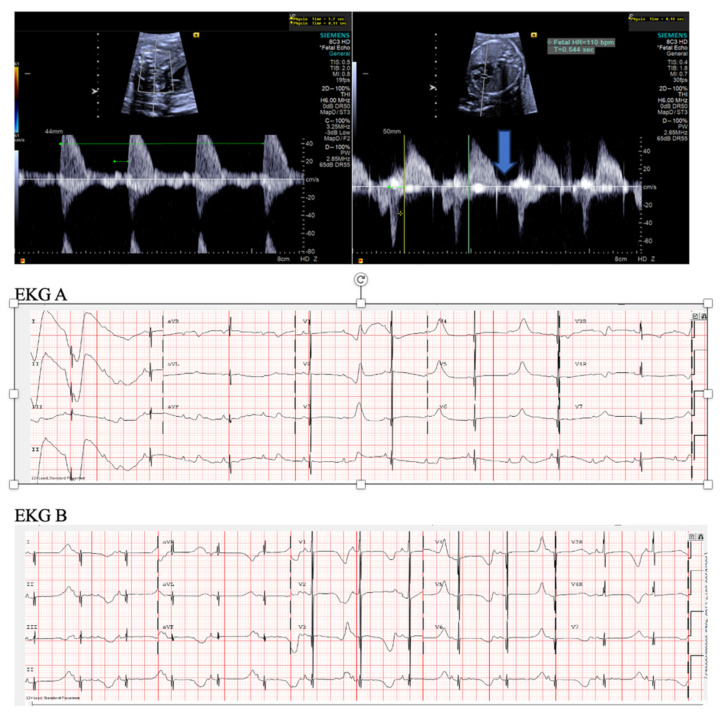
Fetal bradycardia (heart rate < 3rd percentile for GA) with a normal mechanical PR interval but a prolonged isovolumic relaxation time, suggestive of long QT syndrome. Postnatal EKGs confirming the prenatal concern for long QT syndrome. EKG [A]: sinus bradycardia (HR 49), markedly prolonged QT (>800 msec), 2:1 AV block because of marked QT prolongation (prolonged ventricular repolarization). EKG [B]: sinus bradycardia (HR 80 s), marked QT prolongation (~700 msec), T wave alternans (beat-to-beat variability in the repolarization phase of the ventricles and associated with increased risk of ventricular tachyarrhythmias, especially polymorphic ventricular tachycardia (torsade de pointes) and ventricular fibrillation). Genetic testing revealed a VUS in CALM2 (D130N). While this variant has not been previously reported, other mutations at the same site (D130G and D130V) have been reported in association with LQTS with 2:1 AVB. De novo calmodulin mutations have been found in children with LQTS with extreme QT prolongation, negative family history, and early childhood cardiac arrest.

Long QT syndrome may present in utero with sinus bradycardia (most commonly) and prolongation of the left ventricular isovolumic relaxation time [75], functional 2°-AV block (because repolarization is so prolonged, atrial contraction initiates before ventricular repolarization ends), and ventricular tachycardia/torsades de pointes, characterized by variable V-V intervals during tachycardia and changing ventricular outflow Doppler amplitudes [71,76]. It is important to screen for a family history of generalized seizures, recurrent syncope, sudden death, unexplained accidental death from drowning or car accident, unexplained fetal loss, fetal sinus bradycardia, neural deafness, or syndactyly [64,66]. De novo variants (Figure 9), especially in SCN5A, have been strongly associated with complex arrhythmias (AV block with 3:1 conduction, QRS alternans in 2:1 AV block, slow monomorphic ventricular tachycardia, and long-cycle length torsades) and perinatal death [77]. Rare heritable bradycardia syndromes, including those due to SCN5A (LQTS3 and Brugada syndrome), HCN4 (familial sick sinus syndrome), CACNA1D, RYR2, CASQ2, MYH6, and ANK2, may present with low atrial or junctional bradycardia [9,78,79].

**Figure 10:**
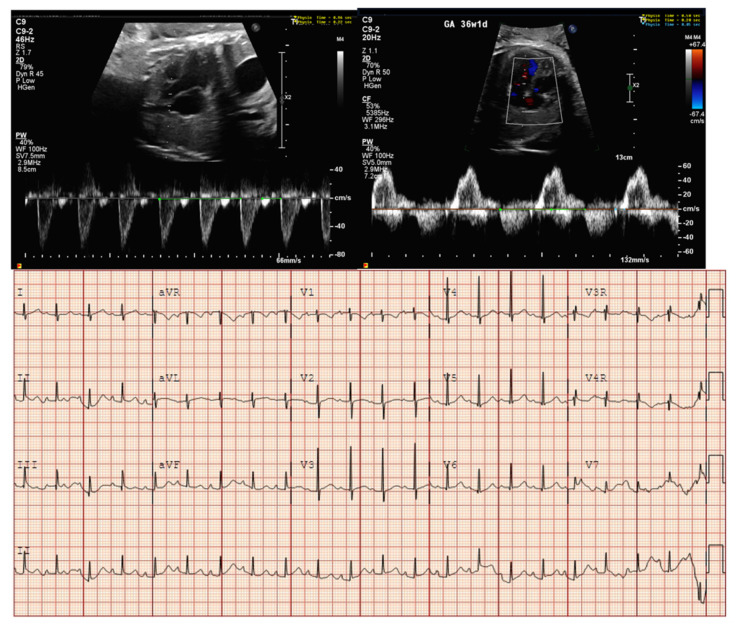
Fetal bradycardia (heart rate < 3rd percentile for GA) with normal mechanical PR interval and prolonged isovolumic relaxation time; postnatal EKG confirming LQTS. The fetal diagnosis led to diagnosis of LQTS in her asymptomatic mother.

Fetal diagnosis of LQTS and other channelopathies can decrease the risk for intrauterine fetal demise and premature delivery (sinus bradycardia or ventricular bradycardia due to functional 2°-AV block is often misinterpreted as fetal distress), can allow for fetal therapy (even the hydropic fetus with ventricular tachycardia can be successfully treated in utero) and maternal avoidance of QTc prolonging medications (oxytocin and ondansetron) to prevent fetal torsades, and can identify affected family members without apparent symptoms (Figure 10) [65]. Ventricular tachycardia associated with LQTS and other channelopathies can be treated in utero with transplacental IV magnesium (first-line therapy), propranolol, lidocaine, and/or mexiletine [66,80].

**Figure 11:**
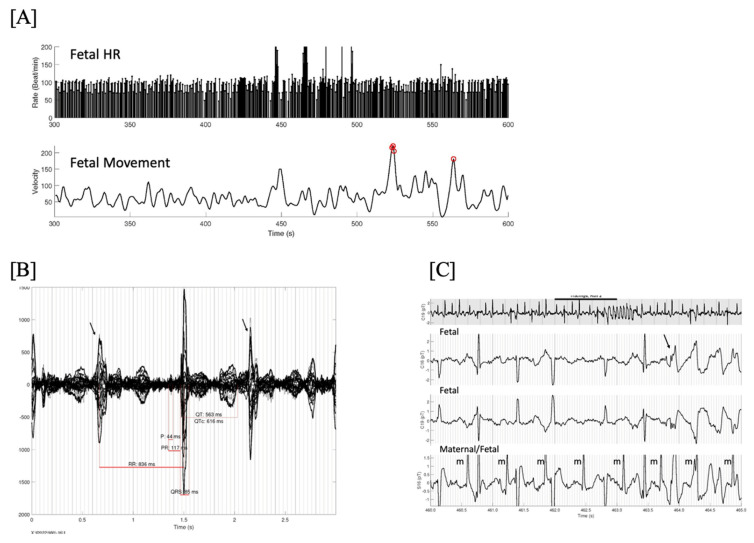
(**A**) Fetal HR trends over 5 min showing several brief episodes of torsades de pointes (TdP) at a rate exceeding 200/min, and very frequent baseline ectopy and bradycardia. Line 2 is the simultaneous fetal actogram. Red circles are times of significant fetal movement, which do not appear to coincide with the TdP. One of these episodes at 460–465 s is shown in (**C**). (**B**) Signal-averaged fMCG showing marked QTc prolongation and late-coupled PVCs (arrows). (**C**) 25-s rhythm strip in grey. Below it are 3 simultaneous channels (2 fetal, 1 maternal/fetal) from the mid-portion (black line, 5 s), demonstrating the initiation of TdP due to a late-coupled PVC (arrow). Note the markedly prolonged QTc and intermittent 2nd degree AV block. This fetus was not recognized to have TdP by echocardiography due to the brief and intermittent nature of the TdP. One of the advantages of fMCG is that by continuously recording beat-to-beat, these short bursts of tachycardia can be recognized, providing a better risk stratification. In this case, the TdP was aggravated by maternal Vitamin D deficiency. Treatment with IV magnesium, propranolol, and vitamin D stabilized the TdP. The baby underwent genetic testing at birth and was found to have the maternal pathogenic KCNH2 variant.

A management approach to fetal sinus bradycardia and suspected channelopathy, includes periodic echocardiograms (every 2 weeks × 2 and then every 4 weeks until delivery), home fetal heart rate monitoring (2–3 times daily to detect 2:1 AV block or torsades), periodic biophysical profiles (especially to assess the baseline fetal heart rate prior to labor and delivery), and fetal magnetocardiography (fMCG) to measure the QTc and evaluate for late premature ventricular contractions or torsades (Figure 11) [66,76,77]. It also important to obtain parental EKGs, review the maternal medication list for any QT prolonging drugs, and correct low maternal calcium, magnesium, and 25-OH-vitamin D levels [2]. No fetal therapy, but observation is recommended, for sinus or low atrial bradycardia [2]. Delivery at a tertiary care center is recommended if the QTc on fMCG is >580 msec, if there have been torsades or persistent 2:1 AV block, and if cardiac pacing may be needed postnatally. EKG, 24 h telemetry monitoring, and evaluation by a pediatric cardiologist/electrophysiologist are important aspects of postnatal care, with postnatal genetic testing for LQTS, Brugada syndrome, and other channelopathies [2]. Cascade genetic testing is recommended for parents and first-degree relatives of positive cases [9]. Because of the very significant association between fetal sinus bradycardia and channelopathies, Chaudhry-Waterman et al. have recommended genetic testing for infants with a history of FHR < 3rd percentile for gestational age even if the postnatal ECG demonstrated a normal QTc interval [9]; some experts also advocate for amniocentesis and prenatal genetic testing to help guide fetal therapy and to determine familial risk when the low risk of amniocentesis is determined to be negligible compared to the knowledge gained [81,82].

### 5.2. Anti-Ro/SSA Mediated Heart Block

**Figure 12:**
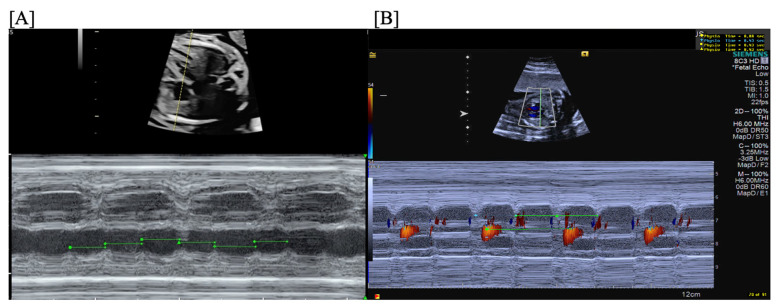
Fetal third-degree AVB demonstrated by traditional m-mode (**A**) and confirmed by color m-mode (**B**) (m-mode through the atrium provides the atrial rate with overlaid color Doppler demonstrating the ventricular rate).

**Figure 13:**
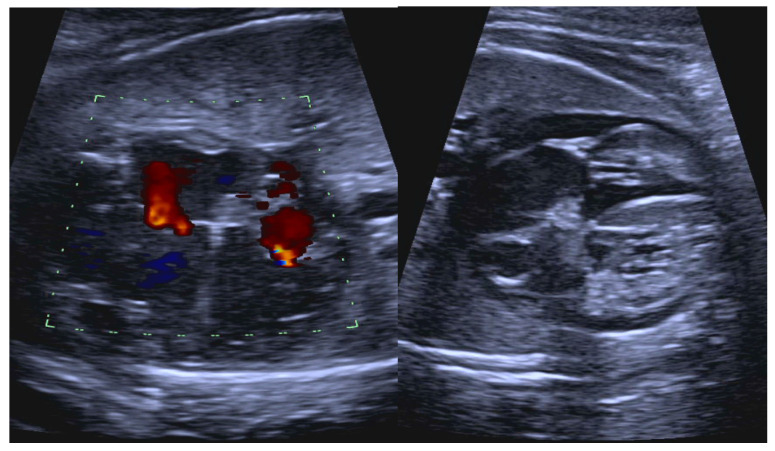
Fetal anti-Ro/SSA-mediated carditis characterized by valvar insufficiency, ventricular dilation, increased endocardial echogenicity, and pericardial effusion.

**Figure 14:**
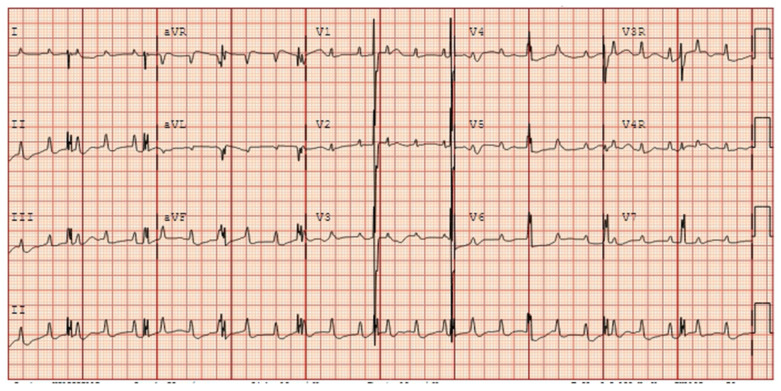
Postnatal EKG in a newborn with prenatally diagnosed anti-Ro/SSA mediated third-degree atrioventricular block.

Fetal cardiac neonatal lupus (C-NL), also known as congenital complete heart block, is a disease of the second trimester associated with maternal anti-Ro/SSA antibodies [83,84,85]. Anti-Ro/SSA antibodies can cross the placenta as early as 11 weeks of gestation and cause inflammation, fibrosis, and calcification of the conduction system leading to AV block, cardiomyopathy, valvulitis, endocardial fibroelastosis, pericardial effusion, and hydrops (Figure 12, Figure 13 and Figure 14); extranodal disease often coincides or heralds the development of AV block but may occur in its absence [83,84,85]. Other arrhythmias that may occur in the presence or absence of AV block include JET, atrial flutter, ventricular arrhythmias, sinus bradycardia/sinus node dysfunction, and QT prolongation [48,86]. Complete, 3°-AV block is irreversible and has significant associated mortality and morbidity, with most (>70%) postnatal survivors requiring permanent cardiac pacing and with a ~10% risk of concomitant cardiomyopathy requiring cardiac transplantation [85,87]. Risk factors for neonatal death include premature delivery (<32 weeks of gestation), low ventricular escape rate (<55 bpm), endocardial fibroelastosis, cardiac dysfunction, and hydrops [88,89].

Prevalence of maternal anti-Ro/SSA antibodies is 0.9% [90]. Fetal C-NL (AV block and/or cardiomyopathy) occurs in ~1:20,000 live births and results in death in one fifth of cases [83,84,89]. The risk of fetal C-NL is 2% unless a previous offspring has been affected, which raises the risk to 18% [83,84,85]. High antibody titers are necessary but not sufficient for fetal C-NL and appear unrelated to maternal rheumatologic symptoms [91,92]. One study found that mothers with hypothyroidism/anti-thyroglobulin antibodies had a 9-fold increased risk of fetal heart block compared to mothers with anti-Ro/SSA antibodies alone [93]. Published data suggests that over 50% of fetuses who develop fetal C-NL are in mothers who were unaware that they have anti-Ro/SSA antibodies [83,84]. 

**Figure 15:**
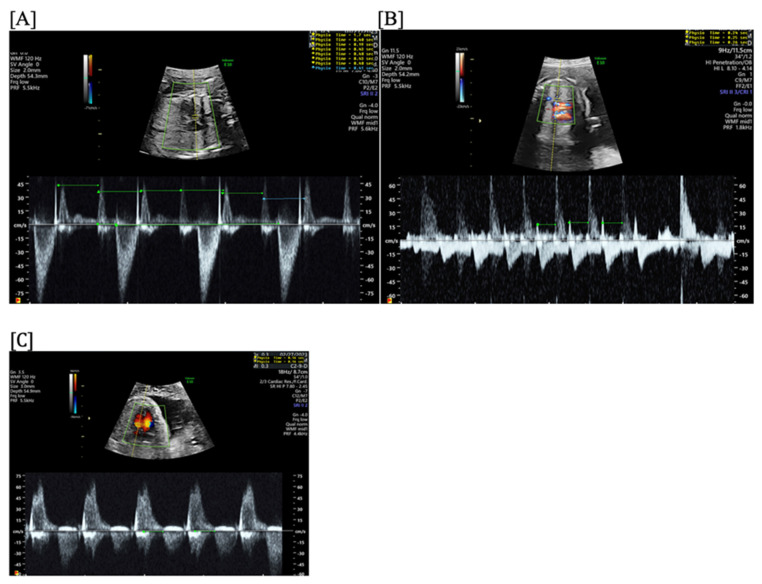
Second-degree atrioventricular block (AVB) with 2:1 AV conduction in a fetus exposed to maternal anti-Ro/SSA antibodies, characterized by a regular atrial rate and 2:1 AV conduction. Irregular rhythm on home fetal heart rate monitoring at 21 4/7 weeks prompted emergent fetal echocardiogram (2 h later), which confirmed second-degree AVB (**A**). After treatment with IVIG (1 g/kg maternal weight, max 70 g) and after initiating treatment with oral dexamethasone (8 mg/day for 10 days; 4 mg/day through 28 6/7 weeks; then 3 mg/day from 29 weeks to 29 6/7 weeks; then 2 mg/day until delivery), the rhythm became type 1 s-degree AVB (Wenckebach) (**B**) within 2 days of therapy and persistent first-degree AVB (**C**) within 1 week of therapy, which persisted until delivery and into infancy.

Transition from sinus rhythm to 3°-AV block or cardiomyopathy usually occurs between 18- and 25-weeks of gestation and can be rapid (<12 h) [94,95]. During this transition period, when the fetus is in 2°-AV block, emerging data suggest that treatment with intravenous immunoglobulin (IVIG, 1 g/kg maternal weight, max 70 g) and oral dexamethasone (8 mg/day for 10 days; 4 mg/day through 28 6/7 weeks; then 3 mg/day from 29 weeks to 29 6/7 weeks; then 2 mg/day until delivery) may halt progression to 3°-AV block and potentially restore normal sinus rhythm (Figure 15) [80,95,96]. Once 3°-AV block develops, treatment with IVIG and dexamethasone will not restore conduction but may improve survival and long-term outcomes by modifying the inflammatory damage to the myocardium, valves, and endocardium and by resolving associated hydrops [96,97,98]. 

Indications for dexamethasone include 1°-AV block with signs of myocardial inflammation, 2°-AV block, 3°-AV block with hydrops or myocardial inflammation, and other arrhythmias (JET, atrial flutter, or ventricular arrhythmias) in the presence or absence of AV block [80,97,98,99]. Potential side effects of dexamethasone, for which some have argued against its use, include fetal growth restriction, oligohydramnios, adrenal suppression, possible learning disabilities/decreased brain growth, and maternal risk glucose intolerance [29,100,101]. Indications for IVIG include 2°-AV block or 3°-AV block with hydrops or myocardial inflammation [97]. Repeat doses 2–4 weeks after the initial dose may be indicated if there are still signs of active cardiac inflammation [96]. β-adrenergic agents (oral terbutaline 5 mg every 6 h) may be used to increase low fetal heart rates (<55 bpm), especially in the setting of cardiac dysfunction or hydrops, but with varying reports of survival benefit; mothers taking terbutaline may experience palpitations necessitating dose reduction [29,101,102]. Low maternal 25(OH)-vitamin D, calcium, and magnesium levels may increase the risk of arrhythmias in fetal C-NL and should be treated [103]. Hydroxychloroquine has been shown to reduce the recurrence risk of fetal AV block by ~50% in mothers who have had a child previously affected by anti-Ro/SSA mediated fetal C-NL; this drug, started before 10 weeks of gestation, is recommended for secondary prevention of fetal cardiac disease in anti-SSA/Ro-exposed pregnancies [104].

Risk stratification through maternal anti-Ro/SSA titer levels and home surveillance using fetal handheld Doppler monitors are now increasingly being used by providers caring for anti-Ro/SSA positive pregnancies [105]. In 4 cases of 2°-AV block identified by home fetal heart rate monitoring and treated with dexamethasone and IVIG in <12 h, AV block reversed [95]. This data, as well as other studies evaluating the use of dexamethasone and IVIG in modulating disease severity in fetuses and infants with C-NL, recognizes the need to identify and treat disease expediently to improve outcomes [80,95,96]. Fetuses/infants who present with 3°-AV block because of unknown maternal anti-Ro/SSA antibody status are unlikely to have the same benefits of therapy as fetuses/infants whose conduction and/or extranodal diseases are identified and treated early and rapidly [83,98]. 

According to the 2014 American Heart Association Scientific Statement on the Diagnosis and Treatment of Fetal Cardiac Disease, fetal echocardiographic screening of anti-Ro/SSA positive pregnancies is recommended either weekly or biweekly to surveil for development of fetal C-NL [29]. A statement from the Society for Maternal-Fetal Medicine in 2023 on systemic lupus erythematosus in pregnancy challenged the need for fetal echocardiographic screening for assessment of mechanical PR intervals and the benefit of routine steroid use to treat fetal AV block [106]. In 2020, investigators launched the NIH-supported prospective study, “Surveillance and Treatment to Prevent Fetal Atrioventricular Block Likely to Occur Quickly (STOP BLOQ; ClinicalTrials.gov: NCT04474223)”, to optimize the likelihood of timely detection of fetal C-NL using home fetal heart rate monitoring and institute early treatment when indicated. The SLOW HEART REGISTRY of Fetal Immune-mediated High-Degree Heart Block (ClinicalTrials.gov: NCT04559425) also launched in 2020 as a multi-center, observational study to prospectively compare outcomes through the first 3 years of life between steroid-treated and untreated fetuses with anti-Ro/SSA mediated 2°- or 3°-AV block.

The prognosis for children with heart block is generally good, but in some cases the condition is fatal. Overall, older studies have reported a 10–30% perinatal mortality (with 31% of deaths occurring in utero and 68% in the first year) [83,84,92]. Survivors with 3°-AV block require permanent pacing (at least 50% in the first year after birth) [89]. Additionally, there is a long-term risk of developing dilated cardiomyopathy (5–23%, and can occur pre- or postnatally), and/or requiring a heart transplantation [83,107]. However, a recent study by Mawad et al. reported a low risk of perinatal mortality (95% fetal and 93% neonatal survival) and postnatal cardiomyopathy in 130 fetuses who received transplacental dexamethasone from the time of diagnosis of C-NL (started at a median age of 22.4 weeks of gestation); co-treatment with IVIG improved neonatal survival to 100% [96]. These authors reported that the following variables at C-NL diagnosis were associated with perinatal death: “atrial rate < 90 bpm (odds ratio (OR), 258.4; 95% CI, 11.5–5798.9; *p* < 0.001), endocardial fibroelastosis (OR, 28.9; 95% CI, 1.6–521.7; *p* < 0.001), fetal hydrops (OR, 25.5; 95% CI, 4.4–145.3; *p* < 0.001), ventricular dysfunction (OR, 7.6; 95% CI, 1.5–39.4; *p* = 0.03), and a ventricular rate < 45 beats per minute (OR, 12.9; 95% CI, 1.75–95.8; *p* = 0.034)” [96]. They concluded that “routine transplacental treatment of cardiac NLE (C-NL), including the use of steroids ± IVIG ± a β-adrenergic agent, reduces the risk of perinatal death and the postnatal development of DCM (dilated cardiomyopathy); although fetal growth restriction was observed in 30% of their patients, it did not affect age at delivery, postnatal survival, or neurodevelopmental outcomes [96]. 

C-section delivery at a tertiary care center is recommended in the setting of 3°-AV block, because fetal heart rate changes/variability during vaginal labor and delivery cannot be monitored. The pediatric electrophysiology and cardiothoracic surgery teams should be included in delivery planning in case emergent pacing is needed, especially if there is hydrops or FHR < 60 bpm [29]. 

Because of the perinatal morbidity and mortality associated with fetal heart block, attempts were first made at in utero pacing for 3°-AV block in 1986 [108]; efforts to develop fetal pacing continue. Ongoing research seeks to improve the technology of in utero pacing by developing specially-designed artificial pacing systems, including fully implantable micro-pacemakers [109], and by determining the optimal route for placement of these systems, including fetoscopic methods [110]. Choi et al. are investigating biological technologies that implant engineered tissue into the fetal heart to create an alternative atrioventricular conduction pathway [111]. In 2023, Dr. Charles Berul et al. reported successful use of a modified implantable pulse generator utilizing the technology of the Medtronic Micra^TM^ leadless pacing system to provide cardiac pacing for five small, premature neonates (EGA at birth 28.5–36 weeks; 1.35–2.68 kg weight at pacemaker implantation) [112].

Certainly, variation in prenatal surveillance and management of anti-Ro/SSA antibody positive pregnancies and in postnatal care strategies exists [113]. The ongoing work of multiple investigators in fetal cardiology, rheumatology, obstetrics, maternal–fetal medicine, and electrophysiology seeks to clarify monitoring and treatment recommendations for both mothers and fetuses affected by anti-Ro/SSA antibodies.

## 6. Diagnostic Tools

Technologies for diagnosis and managing pregnancies complicated by fetal arrhythmias continue to evolve in the current era. Fetal cardiotocography, a continuous recording of the fetal heart rate using an ultrasound transducer placed on the mother’s abdomen, has been widely used to assess fetal well-being during labor since the 1960s [6]. This technology is also widely used to perform non-stress testing after 28–32 weeks of gestation to monitor high-risk pregnancies, including those complicated by fetal arrhythmias [6]. A pressure transducer evaluates fetal movement, and a Doppler transducer monitors fetal heart rates. The Monica AN24 is a portable device for monitoring fetal heart rate and uterine activity; it has the highest signal success in the second trimester and lowest in the early third trimester [114]. This technology is also being used to measure fetal cardiac time intervals with variable success [115]. 

Fetal heart rate auscultation by handheld Doppler is routinely used in the obstetrical office to assess fetal heart rate as a marker for well-being. The Fetal Heart Sounds at Home study demonstrated the feasibility of using home fetal heart rate monitoring to identify heart rate and rhythm irregularities in mothers with anti-Ro antibodies, which was empowering to the mothers [51,116].

Fetal electrocardiography (fECG) historically suffers from a low signal to noise ratio with difficulty distinguishing the low-amplitude fetal electrocardiographic signal from the maternal signal [2]; fetal movement, fetal orientation, maternal factors, and environmental noise influence signal processing. In 2020, Sethi, et al. reported successful use of a noninvasive fECG device with blind source separation (IRIS^TM^; Atom Medical Company, Tokyo, Japan) in three cases of clinically suspected LQTS [117]. Their fECG system had high diagnostic accuracy compared to current gold-standard tests of fMCG and postnatal testing [117]. More recently, the group from Children’s National described a new frequency-based technique for attenuating the maternal ECG to enhance and extract the fECG [118]. Ongoing efforts utilize artificial intelligence, including artificial neural networks and deep learning models, to detect fetal arrhythmias [119,120,121,122].

Echocardiography is the primary tool for diagnosing fetal arrhythmias. M-mode (through atria and ventricles) and pulse wave Doppler (left ventricular inflow and outflow, SVC and ascending aorta/innominate vein and transverse aorta, pulmonary artery and pulmonary vein) are used to determine the atrial and ventricular rates, the relationship of atrial and ventricular contractions, and the onset and termination of the arrhythmia [14,123,124,125,126]. The challenge of using ultrasound to diagnose fetal arrhythmias is that electrical activity must be inferred from mechanical events [14]. Atrial and ventricular wall motion (M-mode) or venous and atrial flow signals (pulse Doppler) are used to define atrial (P wave) and ventricular (QRS wave) depolarization [14], but electromechanical dysfunction has been reported with several types of arrhythmias, including atrial flutter (AF) [2]. Intermittent myocardial akinesia or hypokinesia can mimic absence of P or QRS waves when they are truly present [2]. When atrial and ventricular contractions occur simultaneously (as in AV block), the atrial contraction can be missed when the atrioventricular valve is closed [127]. Mechanical rhythm then does not always accurately reflect the electrical rhythm. Fetal position, activity, and image resolution can also limit echocardiographic assessment of fetal rhythm. When simultaneous assessment of atrial and ventricular motion cannot be made by traditional M-mode, color M-mode allows the aortic outflow Doppler signal to overly the M-mode atrial signal; mechanical and flow events are displayed simultaneously [127]. This technique can be especially helpful in understanding atrial and ventricular relationships in tachycardia. Doppler sampling in the ductus venosus is often used to assess fetal well-being, but in the setting of fetal tachycardia, ductus venosus flow patterns can help determine tachycardia mechanism [30,128].

Fetal magnetocardiography (fMCG) is another tool for fetal arrhythmia evaluation, which uses superconducting quantum interference device (SQUID) magnetometers to safely and non-invasively measure magnetic fields generated by electrical currents in the fetal heart [2,129,130]. Fetal MCG recordings capture each of the cardiac time intervals (P, QRS, and T waves, RR, PR, and QT intervals) in almost all fetuses over 24 weeks of gestation, and QRS and RR intervals in most fetuses over 17 weeks of gestation [29,131]. By clearly identifying P, QRS, and T waves, fMCG can accurately distinguish types of fetal arrhythmias, monitor the cardioactive effects of anti-arrhythmic drug therapy (including drug effect or toxicity), quantify fetal heart rate variability, and assess cardiac conduction times [130,132]. Currently, these studies must be performed within magnetically shielded rooms to attenuate magnetic interference from external sources, so fMCG is primarily a research tool and is not widely available [29,132]. Portable optically pumped magnetometers are a promising new technology that have the potential to greatly increase the clinical utilization of fetal magnetocardiography and improve fetal electrophysiologic assessment [133].

## 7. Multidisciplinary Collaboration and the Maternal-Fetal Dyad

Pharmacologic management of fetal arrhythmias remains one of the most successful achievements of fetal intervention. Multidisciplinary collaboration is key to successful diagnosis and treatment as both mother and fetus need expert care [4,5]. In the current era, interdisciplinary cardio-obstetrical teams, including obstetricians, specialists in maternal-fetal medicine, fetal/pediatric cardiologists, electrophysiologists, adult cardiologists with interest in cardio-obstetrical care, neonatologists/pediatricians, geneticists/genetics counselors, nurses/care coordinators, and social workers, support the care of pregnancies affected by fetal arrhythmias [134]. Rheumatologists support the care of mothers and fetuses affected by anti-Ro/SSA antibodies and endocrinologists add expertise in pregnancies affected by thyroid disease or complicated by side effects of amiodarone. Medical physicists, device developers, and bioengineers are also part of the interdisciplinary fetal arrhythmia team, developing new diagnostic and therapeutic technologies and using pharmacologic and electrophysiologic computational modeling to optimize transplacental drug treatments. Experts in pharmacology and pharmacogenomics can provide valuable insight into effective transplacental anti-arrhythmic therapy that limits toxicity to mother and fetus [82]. 

Putra et al. recently reported on pharmacogenomic-guided prenatal drug therapy for a maternal/fetal dyad with a severe fetal arrhythmia secondary to a de novo channelopathy [82]. Their multidisciplinary cardio-obstetrics team collaboratively provided “personalized medicine” to reduce drug toxicity and improve drug efficacy [82]. Individualized care using pharmacogenomics may be part of the future of fetal arrhythmia management. 

It is well known that maternal health influences fetal heart rate and rhythm, including medication effects, thyroid disease (especially Grave’s disease), infection, placental dysfunction, autoimmune diseases (especially Sjogren’s syndrome and lupus), and inherited arrhythmias like LQTS [5]. Importantly, fetal arrhythmias may reveal undiagnosed maternal diseases [5]. Recognizing subtle differences in FHR or rhythm may lead to identification of potentially lethal conduction system abnormalities in the mother [65]. Fetal arrhythmia management in the current era understands the importance of caring for the maternal–fetal dyad and utilizes multidisciplinary clinical expertise and technological advances to do so [5].

## 8. Conclusions

Diagnosis and management of fetal arrhythmias have changed over the past 40–50 years with the advent of new technologies, improvement in therapeutic approaches, and recognition of the impact of subtle heart rate and rhythm differences on maternal and fetal health. As we expand the envelope of fetal intervention in the current era, pharmacologic management of fetal arrhythmias remains one of the most successful achievements in fetal therapy. Working to achieve term, vaginal deliveries involve not only thoughtful prenatal care but also careful and collaborative delivery planning, recognizing the ongoing care that may be needed for both mother and fetus in the perinatal period. Multidisciplinary research, including development of diagnostic and therapeutic technologies; basic science, genetic, and biomarker research; multicenter, prospective therapy trials; and registries and consortiums, continues to inform and improve the clinical care of mothers and fetuses affected by arrhythmias. Importantly, patience, vigilance, and interdisciplinary collaboration are key to successful diagnosis and treatment of fetal arrhythmias.

## Figures and Tables

**Table 2:** The primary antiarrhythmic agents used for treating fetal supraventricular tachycardia, including standard transplacental doses, EKG effects, and common side effects. mcg, micrograms; mg, milligrams; bid, twice daily; tid, three times daily; ng/mL, nanograms/milliliter; mcg/mL, micrograms/milliliter; AV, atrioventricular; q, every.

	Digoxin	Sotalol	Flecainide	Amiodarone
**Standard Doses** **(Transplacental)**	Loading dose: 375–500 mcg q8 h × 3 doses OR 500 mcg q12 h × 4 doses Maintenance dose: 250–500 mcg bid Goal drug levels: 1.5–2.5 ng/mL *Intramuscular digoxin*	240–480 mg daily (120–160 mg bid or tid) Starting dose with hydrops: 160 mg bid	300 mg daily (100 mg q8 h or 150 mg bid) Goal drug levels: 0.2–1 mcg/mL If using a combination of digoxin and flecainide, decrease digoxin dose by 50%	Loading dose: 600 mg q6–8 h (1800–2400 mg/day) × 2–5 days Maintenance dose: 200–600 mg/day Goal drug levels: 0.7–2.8 mcg/mL If using digoxin or flecainide in combination with amiodarone, decrease doses of digoxin (by 50%) and flecainide. *Intraperitoneal amiodarone*
**EKG Effects**	Sinus bradycardia First- and second-degree AV block, including nocturnal Wenckebach (Mobitz type I second-degree AV block)	QRS widening (intraventricular conduction delay/bundle branch block) QTc prolongation First-degree AV block	QRS widening QTc prolongation First-degree AV block	QRS widening QTc prolongation Wide P waves Sinus bradycardia First-degree AV block
**Side Effects**	nausea, vomiting, fatigue, blurred vision	nausea, fatigue, dizziness, hypotension	headache, dizziness, visual disturbances (double vision)	nausea, visual disturbances, photosensitivity, rash, gait/coordination/movement problems, peripheral neuropathy/paresthesia thrombocytopenia, maternal/fetal thyroid dysfunction, liver dysfunction
